# Development of a Population Pharmacokinetic Model of Levofloxacin in Healthy Adults and Identification of Optimal Dosing Regimens

**DOI:** 10.3390/ph18050621

**Published:** 2025-04-25

**Authors:** Yun-Jung Lee, Gaeun Kang, Dae-Young Zang, Dong-Hwan Lee

**Affiliations:** 1 Department of Physical Medicine and Rehabilitation, Myongji Hospital, Goyang 10475, Republic of Korea; yjlee@mjh.or.kr; 2 Division of Clinical Pharmacology, Chonnam National University Hospital, Gwangju 61469, Republic of Korea; bp00092@cnuh.com; 3 Division of Hematology-Oncology, Department of Internal Medicine, Hallym University Sacred Heart Hospital, Hallym University College of Medicine, Anyang 14066, Republic of Korea; fhdzang@hallym.or.kr; 4 Department of Clinical Pharmacology, Hallym University Sacred Heart Hospital, Hallym University College of Medicine, Anyang 14066, Republic of Korea

**Keywords:** levofloxacin, population pharmacokinetics, creatinine clearance, lean body mass, Monte Carlo simulation

## Abstract

**Background/Objectives**: Levofloxacin dosing guidelines recommend adjustments only when the creatinine clearance (CrCl) is <50 mL/min. We hypothesized that further dose stratification based on CrCl could improve therapeutic outcomes, even when the CrCl ≥ 50 mL/min. This study aimed to develop a population pharmacokinetic (PK) model of levofloxacin in healthy adults and identify optimal dosing regimens. **Methods**: In this prospective, open-label study, 12 healthy adults received a single dose of levofloxacin. Plasma concentrations were measured using liquid chromatography–tandem mass spectrometry. A population PK model was developed with nonlinear mixed-effects modeling, and Monte Carlo simulations were performed to identify optimal dosing regimens. **Results**: A two-compartment model with first-order kinetics best described the levofloxacin PK profiles. The CrCl was associated with a variation in clearance and lean body mass, with a variation in peripheral volume of distribution. Simulations identified optimal regimens, defined as those achieving a probability of target attainment of at least 90% for the target unbound 24-hour area under the curve at steady state to minimum inhibitory concentration ratio (*f*AUC/MIC), which differed by pathogen (≥30 for Gram-positive bacteria; ≥100 for Gram-negative bacteria). For the ratio *f*AUC/MIC ≥ 30 and an MIC of 0.5 mg/L, 500 mg daily was optimal for patients with a CrCl of 50–89 mL/min. For the ratio *f*AUC/MIC ≥ 100, 1000 mg daily was required in the same CrCl range and MIC value. **Conclusions**: The population PK model incorporating CrCl and lean body mass improved the prediction of levofloxacin PKs. Refining current dosing recommendations by incorporating stratified CrCl and MIC values could optimize therapeutic outcomes, particularly for patients with a CrCl ≥ 50 mL/min.

## 1. Introduction

**Figure A1:**
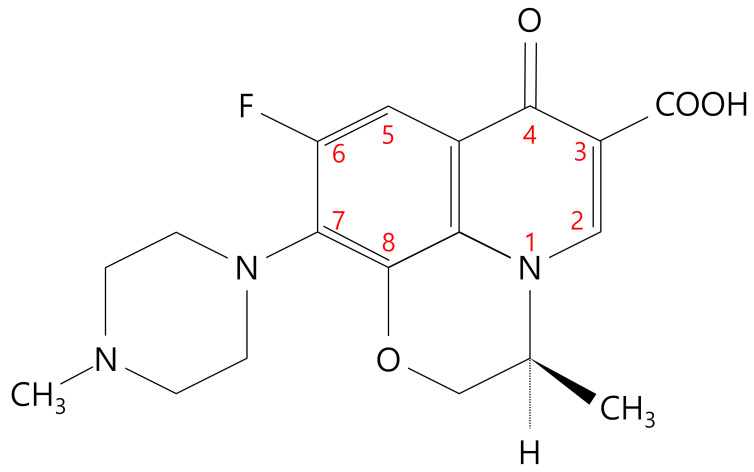
Chemical structure of levofloxacin.

Levofloxacin (LVX), a third-generation fluoroquinolone antibiotic, is critically important for managing various bacterial infections because of its broad-spectrum antibacterial activity. Its spectrum includes Gram-positive and Gram-negative bacteria; atypical organisms such as *Mycoplasma*, *Chlamydia*, and *Legionella*; and various *Mycobacteria species* [1,2]. LVX exhibits nearly 100% bioavailability, allowing effective oral-to-intravenous switch therapy, and demonstrates excellent tissue penetration, making it particularly valuable in treating respiratory, urinary tract, and skin infections [3,4]. The high bioavailability of LVX is largely attributed to its molecular structure, particularly the fluorine atom at the C-6 position and the N-methylated piperazine ring at the C-7 position. These functional groups significantly enhance membrane permeability and systemic absorption [5,6]. The structural formula of LVX is shown in Figure A1. The primary pharmacokinetic/pharmacodynamic (PK/PD) index associated with efficacy is the ratio of the 24-hour area under the free drug concentration–time curve to the minimum inhibitory concentration (*f*AUC/MIC) [7,8].

The current dosing guidelines for LVX, as approved by the United States Food and Drug Administration (FDA) and European Medicines Agency (EMA), recommend dose adjustments based on creatinine clearance (CrCl), primarily for patients with impaired renal function (CrCl < 50 mL/min) [5,9]. However, these guidelines prescribe uniform dosing for all patients with a CrCl ≥ 50 mL/min, disregarding potential variations in drug clearance associated with augmented renal clearance (ARC), which is defined as a CrCl >130 mL/min/1.73 m^2^ [10,11,12]. Emerging evidence indicates that patients with ARC—often observed in critically ill, younger, or obese populations—may exhibit increased LVX clearance, resulting in reduced drug exposure and potentially suboptimal clinical outcomes [13,14]. This highlights the necessity for individualized dosing strategies.

Although previous studies have established ratios of *f*AUC/MIC ≥ 30–40 for Gram-positive bacteria and ≥100–125 for Gram-negative pathogens as optimal PK/PD targets [7,15,16], these values represent the minimum thresholds required for efficacy rather than strict target ranges, which contributes to the challenge of implementing them in clinical practice. A major limitation was that, without a validated PK model, reliable AUC estimation in clinical settings required multiple blood samples, which was impractical. Recently, advanced pharmacometric modeling, such as Bayesian-assisted dosing, has been suggested to better achieve individualized therapeutic targets [17,18,19,20,21]. However, these tailored approaches have not yet been widely adopted for LVX dosing.

To address these issues, a Monte Carlo simulation was first conducted using a population PK model developed from healthy adult volunteers to assess whether current guideline-based dosing regimens effectively achieve the PK/PD target for patients with a CrCl ≥ 50 mL/min. Subsequently, a second Monte Carlo simulation was performed to evaluate various daily dosing regimens stratified according to narrower CrCl intervals (10–19, 20–49, 50–89, 90–129, and 130–170 mL/min). We hypothesized that stratifying the CrCl into narrower intervals and adjusting the LVX dosing accordingly would enhance the probability of target attainment (PTA). Ultimately, this study aims to provide evidence-based dosing recommendations that could improve clinical outcomes in patients with diverse renal functional statuses, particularly those with a broad range of near-normal CrCl values.

## 2. Results

### 2.1. Participants

This study enrolled 12 healthy adults (eight females and four males) (Table 1). All participants completed the clinical trial without experiencing any adverse events. The participants had a median age of 35.5 years (age range of 29.0–44.0 years) and a median BMI of 23.5 kg/m^2^ (BMI range of 18.3–28.9 kg/m^2^). Renal function was evaluated by various methods, including CrCl calculated by the Cockcroft–Gault equation and the estimated glomerular filtration rate (eGFR) using the MDRD and CKD-EPI equations. Laboratory variables, including serum protein, albumin, creatinine, and cystatin C, were within the normal range, confirming the participants’ healthy status.

### 2.2. Population PK Analysis

**Figure 1:**
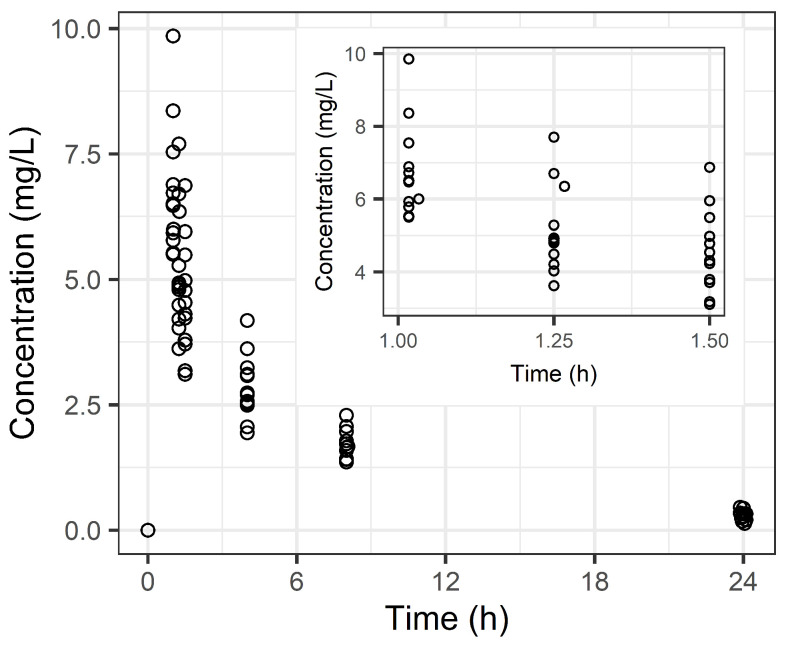
Levofloxacin concentration–time profile in healthy adults. The main plot represents the entire sampling period, and the inset highlights the concentration–time profile during the early phase (1.0–1.5 h). The open circles are observed drug concentrations.

**Figure A2:**
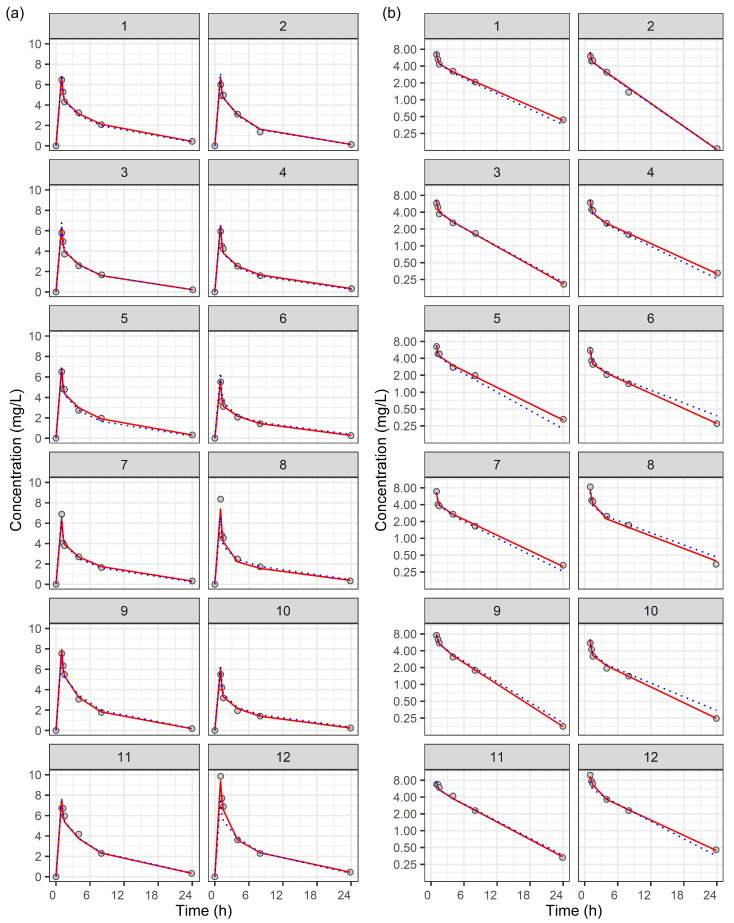
Individual fit plots of levofloxacin: (**a**) normal scale, (**b**) semi-log scale. Closed circles represent observed concentrations, solid red lines indicate individual-predicted concentrations, and dotted blue lines denote population-predicted concentrations.

Eighty-four plasma samples (Figure 1) were analyzed to characterize the PK profile of LVX, which was best described by a two-compartment model (Table 2). Figure A2 presents individual fit plots that illustrate the accuracy of the model in capturing the observed LVX concentrations. The selected two-compartment model included parameters such as the total clearance (CL), volume of distribution in the central compartment (V1), volume of distribution for the peripheral compartments (V2), and intercompartmental clearance between V1 and V2 (Q2). Covariate analysis identified CrCl (affecting CL) and lean body mass (LBM) (affecting V2) as significant factors. In the final model, the covariates CrCl and LBM were incorporated using a power model structure (Table 2). Specifically, CL was modeled as a function of CrCl, and V2 was modeled as a function of LBM. The final model, including both covariates, yielded an objective function value (OFV) of –403.731. The removal of CrCl increased the OFV by 13.996 (to −389.735), and the removal of LBM increased the OFV by 30.237 (to −373.494). Both changes represent a statistically significant improvement in model fit (ΔOFV > 10.83, *p* < 0.001, likelihood ratio test with 1 df), confirming the importance of including these covariates.

**Figure 2:**
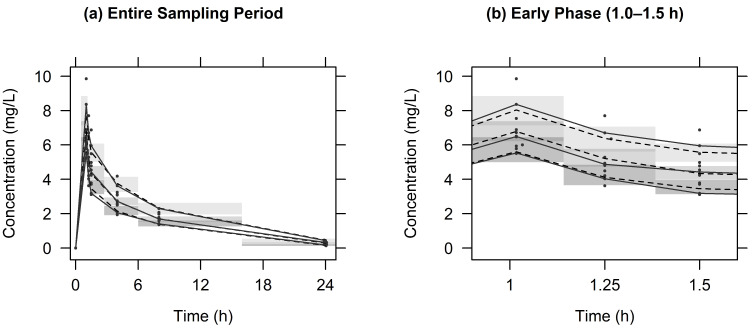
VPC based on simulated concentrations from 1000 virtual datasets of LVX: (**a**) the entire sampling period, (**b**) the early phase (1.0–1.5 h). Closed circles—observed concentrations; solid lines—10th, 50th, and 90th percentiles of observations; dashed lines—10th, 50th, and 90th percentiles of simulated concentrations; shaded areas—95% confidence intervals for the 10th, 50th, and 90th percentiles of simulated concentrations.

**Figure A3:**
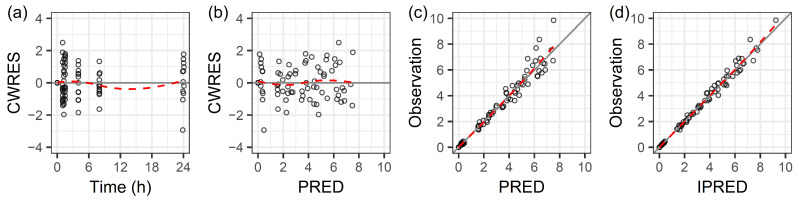
Goodness-of-fit plots for the final PK model of levofloxacin: (**a**) conditional weighted residuals (CWRESs) vs. time, (**b**) CWRESs vs. population-predicted concentration (PRED), (**c**) observed concentration vs. PRED, and (**d**) observed concentration vs. individual-predicted concentration (IPRED). The dashed red lines represent smooth curves.

**Figure A4:**
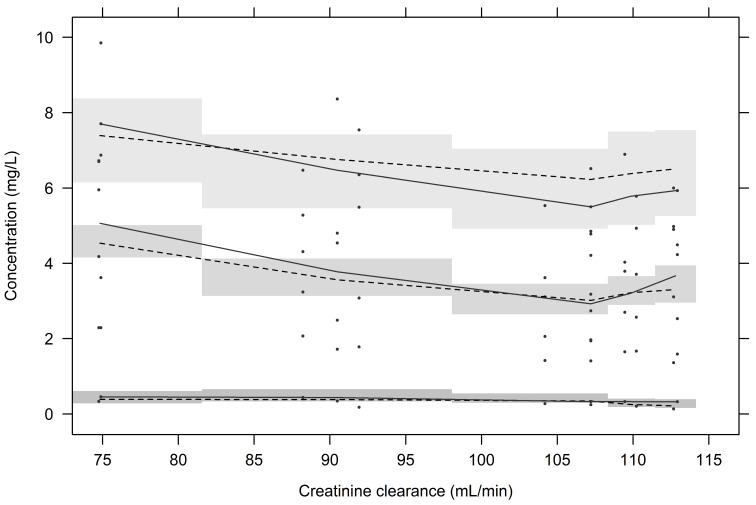
VPC as a function of CrCl based on simulated concentrations from 1000 virtual datasets of LVX: closed circles—observed concentrations; solid lines—10th, 50th, and 90th percentiles of observations; dashed lines—10th, 50th, and 90th percentiles of simulated concentrations; shaded areas—95% confidence intervals for the 10th, 50th, and 90th percentiles of simulated concentrations.

Figure A3 presents diagnostic goodness-of-fit plots for the final PK model of LVX. The conditional weighted residuals (CWRESs) and concentrations were primarily evenly distributed around the x axis or line of identity (y = x), indicating that the structural models were appropriately fitted and unbiased. Figure 2 presents the visual predictive check (VPC) for LVX. The observed 10th, 50th, and 90th percentiles were aligned within the 95% CIs of the corresponding simulated percentiles, confirming that the final PK models accurately reflected the observed drug concentrations and exhibited strong predictive capabilities. Figure A4 shows the VPC as a function of the CrCl. Similar to Figure 2, the observed percentiles were well captured by the simulated prediction intervals, supporting the adequacy of the covariate model in describing the relationship between CrCl and the LVK PK model.

### 2.3. Dosage Simulations

**Figure 3:**
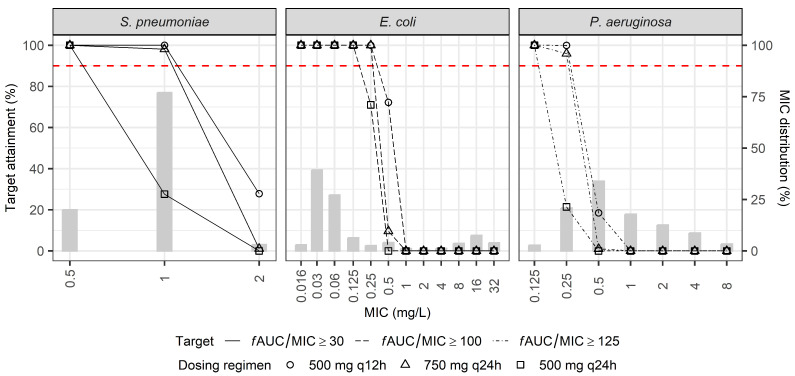
PTA of current guideline-recommended levofloxacin dosing regimens (500 mg q12 h, 750 mg q24 h, and 500 mg q24 h) for patients with CrCl ≥ 50 mL/min. The PTA was evaluated at pathogen-specific PK/PD targets: *f*AUC/MIC ≥ 30 for *Streptococcus pneumoniae*, *f*AUC/MIC ≥ 100 for *Escherichia coli*, and *f*AUC/MIC ≥ 125 for *Pseudomonas aeruginosa*. Gray bars indicate MIC distribution of respective pathogens. The dashed red horizontal lines indicate the PTA threshold of 90%.

In the first simulation, the current dose regimens in patients with normal CrCl were assessed to achieve a PTA of at least 90% for the following PK/PD indices: *f*AUC/MIC ≥ 30 for *Streptococcus pneumoniae*, ≥100 for *Escherichia coli*, and ≥125 for *Pseudomonas aeruginosa* (Figure 3). All dosing regimens met the target attainment criteria at low MIC values; however, the PTA significantly decreased at higher MIC values. For *S. pneumoniae*, the PTA dropped notably at MIC values ≥ 2 mg/L, particularly with the 500 mg q24h regimen. For *E. coli* and *P. aeruginosa*, the PTA sharply declined at MIC values ≥ 0.5 mg/L across all tested dosing regimens, suggesting the limited effectiveness of current standard doses against pathogens with elevated MICs and highlighting the potential need for more precise CrCl-based dosing adjustments.

**Figure 4:**
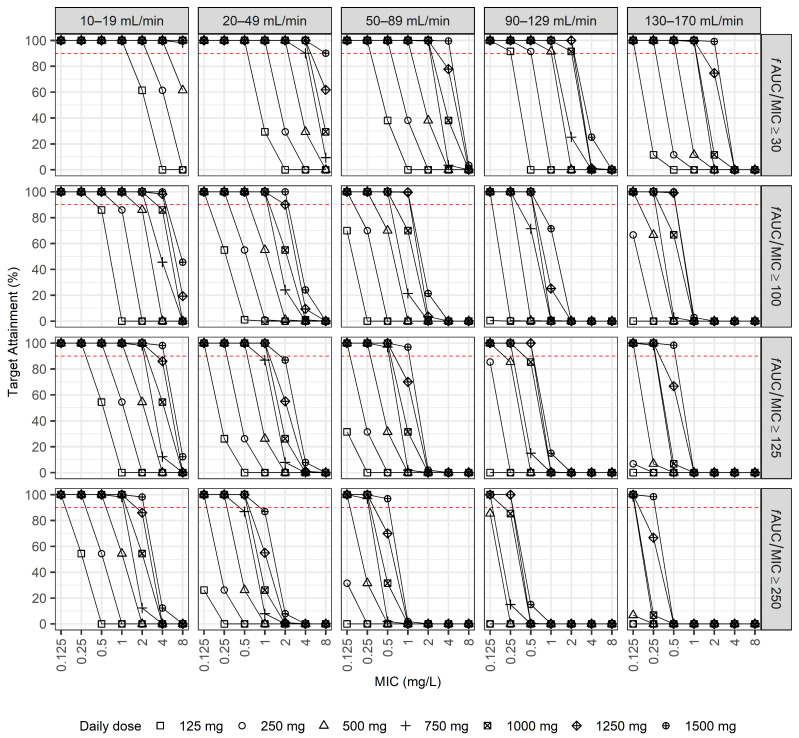
PTA across various levofloxacin daily doses stratified according to narrower CrCl intervals (CrCl: 10–19, 20–49, 50–89, 90–129, and 130–170 mL/min). Simulations were performed at four PK/PD targets (*f*AUC/MIC ≥ 30, 100, 125, and 250) across different MIC values. The dashed red horizontal lines indicate the PTA threshold of 90%.

In the second simulation, the PTA was evaluated for various daily doses of LVX across narrower CrCl intervals (i.e., CrCl: 10–19, 20–49, 50–89, 90–129, and 130–170 mL/min), considering four PK/PD targets (i.e., *f*AUC/MIC: ≥30, 100, 125, and 250) (Figure 4). For the lowest PK/PD target (*f*AUC/MIC ≥ 30), lower doses (125–500 mg/day) generally provided adequate PTAs (≥90%) for pathogens with MIC values ≤0.5 mg/L, particularly in patients with CrCl rates between 10 and 49 mL/min. However, as the CrCl increased to ≥50 mL/min, higher daily doses (≥750 mg/day) were necessary to maintain adequate PTAs, particularly for pathogens with MIC values exceeding 0.5 mg/L. For intermediate targets (*f*AUC/MIC ≥ 100 and ≥125), achieving an adequate PTA was more challenging. In patients with a CrCl ≥ 50 mL/min, doses of at least 1000 mg/day were required to achieve a PTA of ≥90% at MIC values of 0.25–0.5 mg/L. Even with the highest simulated doses (1250–1500 mg/day), the PTA significantly declined at MIC values ≥ 1 mg/L, especially in those with ARC. At the highest PK/PD target (*f*AUC/MIC ≥ 250), achieving a PTA ≥ 90% was challenging across all CrCl categories. Only patients with substantially reduced CrCl (10–49 mL/min) achieved adequate PTAs at lower MIC values (≤0.5 mg/L) when higher dosing regimens (≥750 mg/day) were used. For patients with a CrCl ≥ 90 mL/min, including those with ARC, even the highest simulated doses (1500 mg/day) were insufficient to consistently meet therapeutic targets at MIC values >0.5 mg/L. These findings underscore the need for more precise, CrCl-based dosing strategies, particularly for patients with infections involving pathogens with higher MIC values, to ensure optimal therapeutic outcomes.

Table 3 summarizes the recommended LVX dosing regimens derived from the second simulation based on CrCl categories and pathogen-specific MIC values to achieve a PTA ≥ 90% at four PK/PD targets (*f*AUC/MIC ≥ 30, 100, 125, and 250). Patients with lower CrCl rates generally required lower daily doses to achieve therapeutic targets. Conversely, as the CrCl improved, higher daily doses were required to reach the optimal PTA, particularly for patients with elevated MICs. This dosage recommendation enables tailored dosing decisions considering both CrCl and pathogen susceptibility.

## 3. Discussion

In this study, we successfully developed a population PK model of LVX in healthy adults. The model identified CrCl as a significant predictor of LVX clearance and LBM as a covariate affecting the peripheral volume of distribution. Based on previous reports suggesting that the total clearance of renally eliminated drugs may not be fully explained by CrCl alone [24,25], we also evaluated an alternative model including a non-CrCl component of CL in addition to the CrCl-dependent term. However, the estimated non-CrCl component was negligible, and its inclusion did not improve the model fit. Thus, this component was excluded from the final model. Subsequent simulations assessed the appropriateness of current dosing guidelines for patients with CrCl ≥ 50 mL/min and explored alternative regimens stratified by narrower CrCl intervals.

Building upon our population PK model of LVX in healthy adults, we compared our findings with previous studies to contextualize our results (Table 4). When scaled by body size (weight or LBM) and considering the typical CrCl of the study populations, our findings are broadly consistent with previous reports in similar populations [26,27]. The differences observed in patient populations [28,29] underscore the importance of clinical status and covariates, reinforcing the utility of our developed model, which incorporates CrCl and LBM to explain interindividual variability in LVX PK profiles. This comparative analysis supports the reliability of our model and its potential contribution to informing individualized dosing strategies, particularly for patients with a CrCl ≥ 50 mL/min.

Our simulations revealed that although standard LVX doses may achieve adequate PTAs at lower MICs, the PTA significantly decreased at higher MIC values, particularly for pathogens such as *E. coli* and *P. aeruginosa*. These findings are consistent with those of previous studies highlighting the importance of achieving sufficient drug exposure to overcome higher MICs and prevent the emergence of resistance [27,30,31,32]. Current FDA and EMA guidelines primarily recommend dose adjustments for patients with a CrCl < 50 mL/min. However, our simulations revealed that even for patients with a CrCl ≥ 50 mL/min range, finer stratification and dose adjustments based on the CrCl and MIC could be beneficial. Consistent with recent shifts toward personalized antibiotic stewardship [33], including Bayesian-assisted dosing strategies for vancomycin [34], our findings advocate for the adaptation of similar individualized dosing approaches for LVX.

In particular, our simulations suggest that for a target *f*AUC/MIC ≥ 30 and an MIC of 0.5 mg/L, a daily dose of 500 mg is optimal for patients with a CrCl of 50–89 mL/min. However, achieving a higher target (*f*AUC/MIC ≥ 100), often recommended for Gram-negative infections, necessitates a higher dose of 1000 mg/day for the same CrCl group and MIC [18,30]. This highlights the potential inadequacy of the uniform dosing strategy currently recommended for patients with CrCl ≥ 50 mL/min, particularly those infected with pathogens with elevated MIC values.

Furthermore, our study also considered the effects of ARC. In this subgroup, even the highest simulated doses (1500 mg/day) were insufficient to reliably achieve therapeutic targets at MIC values above 0.25 mg/L for higher *f*AUC/MIC targets. This finding aligns with the existing literature showing that standard doses of renally cleared drugs, including LVX, may yield subtherapeutic drug concentrations in patients with ARC, increasing the risk of treatment failure [13,14,35]. This highlights the need for careful consideration of the renal function, potentially including assessment for ARC, when prescribing LVX.

However, despite ongoing efforts to develop population PK models for LVX, widespread clinical adoption of individualized dosing has been limited. This is partly due to the heterogeneity of patient populations across studies—differences in renal function, body composition, disease states, and coadministered therapies often make it difficult to apply existing models beyond the specific groups in which they were developed. As a result, there is a growing need for integrated models built upon pooled data from diverse populations. Although our study was conducted in healthy adults, it contributes to this foundational base by providing high-quality PK data obtained under tightly controlled conditions, free from disease-related confounders such as inflammation, infection, or organ dysfunction. The resulting baseline parameters serve as standardized reference values that help identify and quantify pathophysiological changes in PK profiles when compared with patient data. These data support the interpretation of deviations in drug exposure in clinical populations and inform the design of future PK studies tailored to specific patient groups. For example, comparative analysis between patient-derived PK parameters and our baseline values may reveal the magnitude of PK alterations due to critical illness or comorbid conditions, thus guiding dose optimization strategies in such populations. 

This study has several limitations that should be considered. First, the proposed population PK model was developed from data obtained from healthy volunteers. Thus, the generalizability of the derived PK parameters to other patient populations, such as critically ill or hospitalized patients with infections, may be limited. Although PK data from healthy volunteers may not fully reflect disease-induced alterations, these data provide a critical baseline similar to that from Phase 1 clinical trials, establishing standardized reference parameters free from pathological confounding factors. Such baseline parameters are crucial for identifying and interpreting alterations in drug PKs caused by disease-related physiological changes observed in clinical populations. Consequently, the PK parameters derived from our healthy volunteer cohort should primarily serve as foundational references to guide future PK studies in targeted patient groups, rather than being directly applied to individualized clinical dosing decisions. Second, although theory-based allometric scaling for body size is widely recommended [36,37], our attempts to implement such scaling in this study did not improve the model performance. We explored various body size descriptors, including total body weight, LBM, fat-free mass (FFM), and normal fat mass (NFM). Despite these efforts, the models incorporating allometric scaling did not demonstrate superior fits compared to the final model. We acknowledge that our study’s small sample size and limited variability in body size may have constrained the effectiveness of these scaling approaches. Future studies with larger and more heterogeneous populations are warranted to robustly apply and evaluate theory-based allometric approaches. Third, some dosing simulations in this study involved extrapolated CrCl values beyond the observed range in our dataset. While these scenarios reflect clinically relevant populations with ARC, the predictive performance of the model at these extremes has not been directly validated and should be interpreted with caution. Fourth, our Monte Carlo simulations evaluated LVX doses up to 1500 mg/day, exceeding the currently approved maximum daily doses (FDA: 750 mg/day; EMA: 1000 mg/day). For example, according to our PK model, the LVX clearance is approximately 3.98 L/h when the CrCl is 30 mL/min. With an increase in the CrCl to 120 mL/min, the clearance would proportionally increase approximately fourfold (to approximately 15.3 L/h), necessitating a similarly increased dose (e.g., from 250 mg q12h at 30 mL/min to 1000 mg q12h at 120 mL/min) to maintain an equivalent AUC. Nonetheless, the safety and tolerability of these high-dose regimens have not been thoroughly evaluated in clinical settings. Therefore, prospective clinical validation studies are required to establish the safety, tolerability, and effectiveness of such individualized dosing regimens before their application to clinical practice.

Despite these limitations, our findings provide valuable insights into LVX dosing optimization based on individualized CrCl and pathogen susceptibility. The proposed dosing strategies stratified according to narrower CrCl intervals and MIC values could improve the PTA and potentially enhance clinical outcomes. Further research, including clinical trials involving patient populations with infections and detailed safety evaluations, is warranted to confirm and refine these individualized dosing recommendations. Furthermore, to further optimize the clinical use of LVX, therapeutic drug monitoring strategies should be explored, particularly in patients with ARC or infections caused by pathogens with higher MICs.

To further enhance the clinical applicability of our findings, a user-friendly Shiny app has been developed (available at https://dhlee.shinyapps.io/lvfx/, accessed on 23 April 2025). This tool allows clinicians and researchers to explore the PK profiles and determine appropriate dosage regimens for LVX based on individual patient characteristics and pathogen MIC values, thereby facilitating the implementation of personalized dosing strategies suggested by this research.

In conclusion, our findings provide compelling evidence in favor of a more individualized approach to LVX dosing for patients with a CrCl ≥ 50 mL/min. Incorporating both CrCl and pathogen susceptibility into dosing decisions may improve therapeutic outcomes and should be considered for integration into clinical guidelines.

## 4. Materials and Methods

### 4.1. Participants

This study was approved by the Institutional Review Board of Hallym University Sacred Heart Hospital (IRB No. 2024-05-015) and was conducted according to the principles outlined in the Declaration of Helsinki. The study was conducted at the Clinical Trial Center of the same institution located in Anyang, Republic of Korea, between August 2024 and September 2024. The inclusion criteria were as follows: (1) individuals aged 19 to 55 years at the time of screening; (2) individuals without any history of congenital or chronic health conditions, as determined by a comprehensive medical assessment; and (3) individuals who met the criteria based on thorough health screenings. These screenings encompassed a detailed medical history, vital signs assessment, physical examinations, hematological and biochemical analyses of blood samples, urinalysis, and supplementary diagnostic procedures as deemed necessary. The exclusion criteria were as follows: (1) individuals with a history of clinically significant medical conditions or disorders affecting various body systems, including but not limited to gastrointestinal, cardiovascular, respiratory, endocrine, hepatobiliary, hematologic–oncologic, musculoskeletal, renal, neurological, psychiatric, immunological, urological, ophthalmological, otolaryngological, or genetic disorders; (2) individuals with pre-existing health issues that could influence the PK outcomes of the study drug, such as liver or kidney diseases; (3) individuals with known allergies or previous adverse reactions to meropenem; (4) individuals with positive results for hepatitis B surface antigen, hepatitis C virus antibodies, HIV antigen or antibodies, or syphilis; and (5) women who were pregnant, were nursing, or had the potential to become pregnant.

### 4.2. Study Design

A single dose of 500 mg of LVX was administered to each participant via a 1-h intravenous infusion using an infusion pump. The LVX was dissolved in 100 mL of normal saline. Serial venous blood samples (6 mL each) were collected into heparinized vacutainer tubes at predetermined intervals: immediately before infusion initiation (0 min) and at 61 min, 75 min, 90 min, 4 h, 8 h, and 24 h post-infusion initiation. Following collection, the blood samples were centrifuged at 3000 rpm for 10 min under refrigerated conditions (2–4 °C). The resulting plasma was then separated and transferred into 1 mL aliquots into two cryogenic tubes, which were subsequently stored at ≤−70 °C until analysis.

### 4.3. Drug Assay

Plasma LVX concentrations were quantified using a validated liquid chromatography–tandem mass spectrometry (LC-MS/MS) system with electrospray ionization (ESI). Chromatographic separation utilized a Gemini C18 analytical column (3 μm, 50 × 2.0 mm, Phenomenex Inc., Torrance, CA, USA), employing a gradient mobile phase composed of water containing 5 mM ammonium acetate and 0.1% formic acid (mobile phase A) and acetonitrile with 0.1% formic acid (mobile phase B). Plasma samples were spiked with enrofloxacin as an internal standard and underwent protein precipitation with acetonitrile. The resulting supernatant was diluted with 50% methanol, and a 1 μL aliquot was subsequently injected into the LC-MS/MS system for quantification. The calibration curves exhibited linearity between 0.05 and 50 mg/L, with analytical validation confirming acceptable accuracy and precision.

### 4.4. Population PK Analysis

The population PK analysis of LVX was conducted using a nonlinear mixed-effects modeling technique with NONMEM software (version 7.5, ICON Clinical Research LLC, North Wales, PA, USA). The first-order conditional estimation method with interaction was utilized to estimate both fixed and random effects in the PK parameters, accounting for interindividual variability and residual unexplained variability. To model the PK profiles of LVX, one-compartment (ADVAN1 TRANS2), two-compartment (ADVAN3 TRANS4), and three-compartment (ADVAN11 TRANS4) models were evaluated, each utilizing first-order kinetics, except for the zero-order infusion component.

Model selection and evaluation were guided by several criteria, including the OFV from NONMEM, the precision of parameter estimates (assessed by relative standard errors), IIV shrinkage, diagnostic fit plots, VPCs, and bootstrap analysis. VPCs were generated as a function of both time and CrCl to evaluate the model’s predictive performance across different dimensions. For comparisons of nested models, a statistically significant improvement was defined as a decrease in OFV (ΔOFV) greater than 3.84 (for one degree of freedom [df]) or 5.99 (for two df), corresponding to a *p*-value of <0.05 based on the chi-square distribution. Diagnostic plots for model evaluation included conditional weighted residuals (CWRESs) vs. time and population predictions (PREDs), as well as plots of observed concentrations vs. both PREDs and individual predictions. VPCs were performed by comparing the observed concentrations to the 80% prediction intervals generated from 1000 simulations based on the final PK model. The robustness of the final model was further assessed by calculating the median and 95% confidence intervals (95% CIs) for the PK parameters from 2000 bootstrap samples.

Potential covariates included demographic variables (e.g., age, sex, body weight, height, body surface area, body mass index, LBM, FFM, and NFM) and laboratory measurements (e.g., serum albumin, creatinine, cystatin C, and total protein). LBM was calculated using the James equation [22], which incorporates sex, weight, and height. FFM was estimated using the equation developed by Janmahasatian et al. [38], also based on sex, weight, and height. NFM was calculated using the formula proposed by Anderson et al. [39]: NFM (kg) = FFM + Ffat × (body weight—FFM), where Ffat, a fraction of fat mass, was fixed at 0.211 [40]. CrCl was estimated using the Cockcroft–Gault [41], and eGFR was calculated using the Modification of Diet in Renal Disease [42] and Chronic Kidney Disease Epidemiology [43] equations. Significant covariates influencing PK parameters were identified through a stepwise selection process. A power model structure was used to describe the relationship between covariates and PK parameters. The covariate effect was incorporated using the following form: (individual value/median value)^θ^, where θ represents the estimated exponent. The median value of each covariate in the study population was used as the reference value for scaling. Covariates were included in the model if they resulted in a statistically significant decrease in OFV (*p* < 0.01, ΔOFV < −6.635 for one df) and were excluded if their removal led to a nonsignificant increase in OFV (*p* < 0.001, ΔOFV > 10.83 for one df). Only covariates with both statistical significance and clinical plausibility were considered.

The Perl-speaks-NONMEM (PSN, version 5.5.0, https://uupharmacometrics.github.io/PsN/, accessed on 5 January 2025) toolkit was utilized for covariate identification, model evaluation through VPCs, and nonparametric bootstrapping execution. Post-analysis processing and graphical representation of the results were performed using the R programming environment (version 4.4.3., https://www.r-project.org/, accessed on 2 March 2025).

### 4.5. Dosage Simulations

To test our hypothesis, two Monte Carlo simulation (Appendix B) studies were conducted. The first simulation assessed the appropriateness of current guideline-based dosing recommendations. For this purpose, virtual patients were generated based on demographic characteristics (i.e., age, creatinine, height, weight, and LBM) obtained from normal distributions that reflect the study population. In total, 10,000 virtual patients (5000 males and 5000 females) were simulated to represent healthy CrCl (≥50 mL/min). These virtual patients received dosing regimens currently recommended by FDA and EMA guidelines (500 mg q24h, 750 mg q24h, or 500 mg q12h). PTA was defined as the proportion of simulated individuals achieving the predefined PK/PD targets at steady state: *f*AUC/MIC ≥ 30 for *S. pneumoniae*, ≥100 for *E. coli*, and ≥125 for *P. aeruginosa*. The fraction of unbound drug (*f*) was fixed at 0.7 [1]. MIC distribution data for these bacterial pathogens were obtained from the European Committee on Antimicrobial Susceptibility Testing (EUCAST) database (https://mic.eucast.org/, accessed on 18 February 2025).

In the second simulation, another set of virtual patients (*n* = 1000) was generated to represent different CrCl categories (10–19, 20–49, 50–89, 90–129, and 130–170 mL/min). LVX clearance values were simulated according to the population PK model, and various daily dosing regimens (i.e., 125, 250, 500, 750, 1000, 1250, and 1500 mg) were evaluated. The PTA was calculated for *f*AUC/MIC targets of 30, 100, 125, and 250 across different renal function categories and MIC values. This enabled identification of optimal dosing regimens stratified by CrCl category and MIC.

## Figures and Tables

**Table 1:** Characteristics of the participants.

Variables	Median (Min–Max)		
	Total (*n* = 12)	Female (*n* = 8)	Male (*n* = 4)
Demographic characteristics			
Age, years	35.5 (29.0–44.0)	37.5 (29.0–44.0)	33.0 (30.0–44.0)
TBW, kg	68.0 (47.3–77.2)	58.4 (47.3–75.7)	72.8 (67.5–77.2)
LBM, kg ^a^	47.9 (37.7–60.3)	43.6 (37.7–51.3)	58.1 (53.6–60.3)
Height, cm	165 (152–181)	161 (152–171)	174 (168–181)
Body surface area, m^2 b^	1.75 (1.45–1.94)	1.63 (1.45–1.86)	1.89 (1.77–1.94)
Body mass index, kg/m^2^	23.5 (18.3–28.9)	22.5 (18.3–28.9)	23.8 (21.3–26.8)
Laboratory characteristics			
Protein, g/dL	7.45 (6.80–8.00)	7.50 (6.80–8.00)	7.20 (6.90–7.50)
Albumin, g/dL	4.75 (4.40–5.20)	4.75 (4.40–5.20)	4.80 (4.60–4.90)
Creatinine, mg/dL	0.850 (0.560–1.11)	0.780 (0.560–0.900)	1.06 (0.880–1.11)
Cystatin C, mg/dL	0.880 (0.780–1.06)	0.880 (0.780–1.06)	0.885 (0.850–0.930)
Renal functions			
CrCl, mL/min ^c^	106 (74.8–113)	99.6 (74.8–113)	106 (90.5–113)
eGFR_MDRD_, mL/min/1.73 m^2 d^	80.1 (69.8–121)	81.6 (69.8–121)	78.8 (77.8–99.1)
eGFR_CKD-EPI_CR_, mL/min/1.73 m^2 e^	95.8 (83.1–120)	98.1 (83.1–120)	92.7 (91.6–116)
eGFR_CKD-EPI_CRCC_, mL/min/1.73 m^2 f^	98.2 (81.3–119)	97.2 (81.3–119)	99.4 (94.0–113)

TBW—total body weight; LBM—lean body mass; CrCl—creatinine clearance; eGFR—estimated glomerular filtration rate; MDRD—modification of diet in renal disease; CKD-EPI—chronic kidney disease epidemiology collaboration; CR—creatinine; CC—cystatin C; min—the minimum of (CR or CC)/number and 1; max—the maximum of (CR or CC)/number and 1. ^a^ LBM (female) = 1.07 × TBW − 148 × (TBW/height)^2^ (James formula [22]). LBM (male) = 1.1 × TBW − 128 × (TBW/height)^2^. ^b^ Body surface area = 0.007184 × TBW^0.425^ × height^0.725^ (Du Bois formula [23]). ^c^ CrCl = (140−Age) × TBW/CR × 72 (×0.85 if female). ^d^ eGFR_MDRD_ = 175 × CR^−1.154^ × Age^−0.203^ (×0.742 if female). ^e^ eGFR_CKD-EPI_CR_ (female) = 142 × min (CR/0.7,1)^−0.241^ × max (CR/0.7,1)^−1.200^ × 0.9938^Age^ × 1.012. eGFR _CKD-EPI_CR_ (male) = 142 × min (CR/0.9,1)^−0.302^ × max (CR/0.9,1)^−1.200^ × 0.9938^Age^. ^f^ eGFR_CKD-EPI_CRCC_ (female) = 135 × min (CR/0.7,1)^−0.219^ × max (CR/0.7,1)^−0.544^ × min (CC/0.8,1)^0.323^ × max (CC/0.8,1)^−0.778^ × 0.9961^Age^ × 0.963. eGFR_CKD-EPI_CRCC_ (male) = 135 × min (CR/0.9,1)^−0.144^ × max (CR/0.9,1)^−0.544^ × min (CYS/0.8,1)^0.323^ × max (CYS/0.8,1)^−0.778^ × 0.9961^Age^.

**Table 2:** Parameter estimates and bootstrap medians (95% CIs) for the final PK model of levofloxacin in 12 healthy adult participants.

Parameter	Estimates	RSE (%) [Shrinkage, %]	Bootstrap Median (95% CI)
Structural model			
CL = θ_1_ × (CrCl/105.71) ^θ2^			
θ_1_ (L/h)	13.4	3.36	13.4 (12.6–14.5)
θ_2_	0.901	16.8	0.900 (0.392–1.26)
V1	34.3	8.93	34.5 (29.6–41.3)
Q	72.8	10.9	72.4 (56.6–86.2)
V2 = θ_3_ × (LBM/47.91) ^θ4^			
θ_3_ (L)	67.7	3.42	67.4 (62.1–71.9)
θ_4_	1.75	12.5	1.76 (1.32–2.27)
Interindividual variability			
CL (%)	8.99	15.3 [3.58]	8.23 (4.65–11.1)
Q (%)	36.0	30.6 [10.2]	35.3 (0.000–53.0)
Residual variability			
Proportional error (%)	6.99	13.8 [7.09]	6.72 (4.68–8.24)

RSE—relative standard error; CI—confidence interval; CL—total clearance; V1—central volume of distribution; V2—volume of distribution for the first peripheral compartment; Q—intercompartmental clearance between V1 and V2; CrCl—individual creatinine clearance estimated using the Cockcroft–Gault equation (mL/min, with 105.71 mL/min used as the median reference value); LBM—individual lean body mass (kg), with 47.91 kg used as the median reference value.

**Table 3:** Recommended daily doses of levofloxacin stratified according to CrCl categories and MIC values for achieving PK/PD targets (*f*AUC/MIC ≥ 30, 100, 125, and 250). Values represent recommended daily doses (mg), with corresponding PTA (%) indicated in parentheses. PTA values ≥ 90% indicate adequate dosing for achieving therapeutic targets. Doses resulting in PTA values < 90% are also displayed for reference purposes.

	CrCl (mL/min)				
MIC (mg/L)	10–19	20–49	50–89	90–129	130–170
*f*AUC/MIC ≥ 30					
0.125	125 (100)	125 (100)	125 (100)	125 (100)	125 (100)
0.25	125 (100)	125 (100)	125 (100)	125 (91.5)	250 (100)
0.5	125 (100)	125 (100)	250 (100)	250 (91.5)	500 (100)
1	125 (100)	250 (100)	500 (100)	500 (91.5)	750 (99.2)
2	250 (100)	500 (100)	750 (99.6)	1000 (91.5)	1500 (99.2)
4	500 (100)	750 (90.1)	1500 (99.6)	1500 (25.2)	–
8	750 (98.2)	1500 (90.1)	–	–	–
*f*AUC/MIC ≥ 100					
0.125	125 (100)	125 (100)	250 (100)	250 (100)	500 (100)
0.25	125 (100)	250 (100)	500 (100)	500 (100)	750 (100)
0.5	250 (100)	500 (100)	750 (100)	1000 (100)	1250 (99.2)
1	500 (100)	750 (100)	1250 (99.6)	1500 (71.5)	–
2	750 (100)	1250 (90.1)	1500 (21.4)	–	–
4	1250 (98.2)	1500 (24.1)	–	–	–
8	1500 (45.6)	–	–	–	–
*f*AUC/MIC ≥ 125					
0.125	125 (100)	125 (100)	250 (100)	500 (100)	500 (100)
0.25	125 (100)	250 (100)	500 (100)	750 (100)	750 (98.4)
0.5	250 (100)	500 (100)	750 (96.9)	1250 (100)	1500 (98.4)
1	500 (100)	1000 (100)	1500 (96.9)	1500 (15.0)	–
2	750 (98.2)	1500 (86.9)	–	–	–
4	1500 (98.2)	–	–	–	–
8	1500 (12.3)	–	–	–	–
*f*AUC/MIC ≥ 250					
0.125	125 (100)	250 (100)	500 (100)	750 (100)	750 (98.4)
0.25	250 (100)	500 (100)	750 (96.9)	1250 (100)	1500 (98.4)
0.5	500 (100)	1000 (100)	1500 (96.9)	1500 (15.0)	–
1	750 (98.2)	1500 (86.9)	–	–	–
2	1500 (98.2)	–	–	–	–
4	1500 (12.3)	–	–	–	–
8	–	–	–	–	–

CrCl—creatinine clearance; MIC—minimum inhibitory concentration; *f*AUC—24-h area under the free drug concentration–time curve.

**Table 4:** Comparison of Levofloxacin PK Parameters Across Studies.

Study [Reference]	Population	CL (L/h) (70 kg)	Vss (L) (70 kg)	CL (L/h) (53 kg LBM)	Vss (L) (53 kg LBM)	CrCl (mL/min)
Our Study	Healthy Korean adults	M: 11.9 F: 11.1	M: 123 F: 93.0	M: 13.2 F: 13.7	M: 118 F: 112	M: 106 F: 99.6
Overholser et al. [26]	Healthy U.S. adults	M: 11.2 F: 10.5	M: 113 F: 91.7	M: 10.6 F: 10.6	M: 106 F: 92.2	M: 105.7 F: 97.0
Cao et al. [27]	Healthy Chinese adults	SDS: 9.32 MDS: 7.79	SDS: 103 MDS: 96.9	NR	NR	SDS: 138.0 MDS: 125.9
Cook et al. [28]	Obese patients	AMB: 11.1 HOS: 3.11	AMB: 49.1 HOS: 35.8	NR	NR	AMB: 87.2 HOS: 82.2
Eloy et al. [29]	Patients with bone and joint infections	5.08	88.0	NR	NR	120.2

CL—clearance; Vss—steady-state volume of distribution; CrCl—creatinine clearance; M—male; F—female; LBM—lean body mass; SDS—single-dose study; MDS—multiple-dose study; AMB—ambulatory; HOS—hospitalized.

## Data Availability

The data presented in this study are available on request from the corresponding author. The data are not publicly available due to legal and ethical reasons.

## Abbreviations

The following abbreviations are used in this manuscript:

LVX	Levofloxacin
PK/PD	pharmacokinetic/pharmacodynamic
*f*AUC/MIC	the ratio of the 24-h area under the free drug concentration–time curve to the minimum inhibitory concentration
FDA	United States Food and Drug Administration
EMA	European Medicines Agency
CrCl	creatinine clearance
PTA	probability of target attainment
LC-MS/MS	liquid chromatography–tandem mass spectrometry
OFV	objective function value
VPC	visual predictive check
CWRES	conditional weighted residuals
PRED	population predictions
EUCAST	European Committee on Antimicrobial Susceptibility Testing
TBW	total body weight
LBM	lean body mass
eGFR	estimated glomerular filtration rate
MDRD	modification of diet in renal disease
CKD-EPI	chronic kidney disease epidemiology collaboration
CR	creatinine
CC	cystatin C
CL	total clearance
V1	volume of distribution in the central compartment
V2	volume of distribution for the peripheral compartments
Q	intercompartmental clearance between V1 and V2
RSE	relative standard error
CI	confidence interval
Vd	volume of distribution

## Appendix B. Monte Carlo Simulation

Monte Carlo simulation was used to generate a virtual population by incorporating variability in the pharmacokinetic parameters based on the final model. For each virtual subject, the AUC values were simulated under various dosing regimens, and the PTA was calculated for predefined PK/PD targets across different MIC values.
